# Low-Density Lipoprotein Receptor Deficiency Attenuates Neuroinflammation through the Induction of Apolipoprotein E

**DOI:** 10.3389/fimmu.2017.01701

**Published:** 2017-11-30

**Authors:** Jo Mailleux, Silke Timmermans, Katherine Nelissen, Jasmine Vanmol, Tim Vanmierlo, Jack van Horssen, Jeroen F. J. Bogie, Jerome J. A. Hendriks

**Affiliations:** ^1^Biomedical Research Institute, Hasselt University, Diepenbeek, Belgium; ^2^Molecular Cell Biology and Immunology, VU University Medical Center, Amsterdam, Netherlands

**Keywords:** neuroinflammation, multiple sclerosis, experimental autoimmune encephalomyelitis, low-density lipoprotein receptor, apolipoprotein E

## Abstract

**Objective:**

We aimed to determine the role of the low-density lipoprotein receptor (LDLr) in neuroinflammation by inducing experimental autoimmune encephalomyelitis (EAE) in *ldlr* knock out mice.

**Methods:**

MOG_35–55_ induced EAE in male and female *ldlr*^−/−^ mice was assessed clinically and histopathologically. Expression of inflammatory mediators and apolipoprotein E (apoE) was investigated by qPCR. Changes in protein levels of apoE and tumor necrosis factor alpha (TNFα) were validated by western blot and ELISA, respectively.

**Results:**

*Ldlr*^−/−^-attenuated EAE disease severity in female, but not in male, EAE mice marked by a reduced proinflammatory cytokine production in the central nervous system of female *ldlr*^−/−^ mice. Macrophages from female *ldlr*^−/−^ mice showed a similar decrease in proinflammatory mediators, an impaired capacity to phagocytose myelin and enhanced secretion of the anti-inflammatory apoE. Interestingly, *apoE/ldlr* double knock out abrogated the beneficial effect of *ldlr* depletion in EAE.

**Conclusion:**

Collectively, we show that *ldlr*^−/−^ reduces EAE disease severity in female but not in male EAE mice, and that this can be explained by increased levels of apoE in female *ldlr*^−/−^ mice. Although the reason for the observed sexual dimorphism remains unclear, our findings show that LDLr and associated apoE levels are involved in neuroinflammatory processes.

## Introduction

Multiple sclerosis (MS) is an inflammatory autoimmune demyelinating disease of the central nervous system (CNS). Infiltrated macrophages and resident microglia are considered the dominant effector cells in MS and its animal model, experimental autoimmune encephalomyelitis (EAE) (1, 2). Effector mechanisms include phagocytosis of myelin and the secretion of inflammatory and toxic mediators (3–7). However, increasing evidence indicates that phagocytes can also acquire a disease-resolving phenotype in the CNS. For instance, we recently defined that ingestion of myelin by macrophages alters the inflammatory phenotype by activating the cholesterol- and fatty acid-sensing nuclear liver X receptors and peroxisome proliferator-activated receptors (8–10). These studies stress the pleiotropic role that phagocytes play in the pathophysiology of MS and indicate that lipid-signaling pathways drive the phenotype of phagocytes in MS lesions.

The impact of cholesterol and its metabolites on the pathophysiology of MS is a topic of interest in MS-research (11–16). In the MS brain, marked alterations in both myelin cholesterol and other lipid metabolites are found (13). Furthermore, plasma and cerebrospinal fluid (oxy)sterol levels are disturbed and closely correlate to neurodegenerative processes (14–17). In addition, we showed that lipoprotein levels and function are altered in relapsing-remitting MS patients (RRMS), which may advance disease progression in these patients (18). More specifically, we found smaller low-density lipoprotein (LDL) particles in RRMS patients compared to healthy controls. Interestingly, the average LDL particle size was smaller in male RRMS patients compared to female RRMS patients, which suggests gender differences in lipid metabolism in MS. LDL size is described to be important for LDL function indicating the lipoprotein may be involved in the pathophysiology of disease. Smaller particles have an increased susceptibility to oxidation and a decreased LDL receptor (LDLr) affinity, which may promote their proinflammatory properties (19–21).

The LDLr is a 160 kDa cell surface glycoprotein that plays a key role in plasma cholesterol homeostasis (22). It binds two physiologically important ligands, apolipoprotein B-100 (apoB) and apolipoprotein E (apoE). The only known ligand for the LDLr in the CNS is apoE since apoB is not synthesized in the CNS and, in contrast to apoE, cannot cross the blood–brain barrier (22). Malfunctioning of the receptor in humans results in familial hypercholesterolemia (FH) and is an important risk factor for cardiovascular disease (23). FH is caused by genetic mutations that directly or indirectly affect the function of the LDLr. It is characterized by defective catabolism of LDL, which results in increased plasma cholesterol concentrations and lipid accumulation in macrophages and other immune cells. This promotes proinflammatory responses, including enhanced NF-κB signaling, inflammasome activation, and increased production of neutrophils and monocytes in the bone marrow and spleen (24–28). The ability of lipoproteins to modulate neuroinflammation has been suggested in recent studies (29, 30). In addition, elevated LDL plasma levels were found to increase neurotoxicity in a mouse model for Alzheimer’s disease (30). The above findings indicate that lipoproteins modulate neuroinflammation and neurodegeneration. However, the direct contribution of LDL and LDLr to these processes remains unclear.

Here, we determined the role of the LDLr in neuroinflammation by using the EAE model and *ldlr* knock out (*ldlr*^−/−^) mice. We show that *ldlr* deficiency reduces disease severity in female, but not in male EAE mice. In line with this finding, the inflammatory burden was significantly lower in the CNS of female *ldlr*^−/−^ mice compared to *ldlr*^−/−^ male mice. Macrophages isolated from female *ldlr*^−/−^ mice show an altered inflammatory profile and an impaired capacity to phagocytose myelin. Interestingly, apoE release was enhanced in macrophages derived from females and the beneficial effect of *ldlr* deficiency in EAE was absent in *apoE/ldlr* double knock out mice. This shows that elevated apoE levels likely attenuate EAE severity in female *ldlr*^−/−^ mice. Collectively, our findings indicate that changes in *ldlr* expression contribute to the progression of neuroinflammatory diseases in a gender-specific manner.

## Materials and Methods

### Animals

*Ldlr*^−/−^ and *apoE*^−^*^/^*^−^ mice on a C57BL/6 background and C57BL/6 wild-type (WT) mice were purchased from Harlan Laboratories. Animals were fed a regular diet and housed in the animal facility of the Biomedical Research Institute of Hasselt University. All experiments were performed according to institutional guidelines and were approved by the ethical committee for animal experiments of Hasselt University.

### Induction of EAE

Eleven-week-old mice were immunized subcutaneously at the base of the tail with 200 µg of recombinant human myelin oligodendrocyte glycoprotein MOG_35–55_ emulsified in 100 µl complete Freund’s adjuvant supplemented with 4 mg/ml of Mycobacterium tuberculosis (H37RA strain) according to manufacturer’s guidelines (Hooke Laboratories, Lawrence, USA). Within 2 h and after 22–26 h, mice were intraperitoneally injected with 0.1 ml pertussis toxin. Immunized mice were weighed and scored daily by following a five-point standardized rating of clinical symptoms: 0, no signs; 1, loss of tail tonus; 2, flaccid tail; 3, hind limb paresis; 4, hind limb paralysis; 5, death. Mice were sacrificed at day 18 (peak) and at day 27 post immunization.

### Peritoneal Macrophage Cultures

Mice were injected intraperitoneally with 1.5 ml phosphate-buffered saline (PBS, Sigma-Aldrich) supplemented with 5 mM ethylenediamine teraacetic acid (EDTA, VWR, Leuven, Belgium). Peritoneal cells were cultured for 3 h in RPMI-1640 medium (Lonza, Verviers, Belgium), enriched with 10% fetal calf serum (FCS; Hyclone, Erembodegem, Belgium), 0.5% penicillin/streptomycin (P/S Invitrogen, Merelbeke, Belgium), in a 24-well plate (5 × 10^4^ cells/well) at 37°C with 5% CO_2_. Cells were stimulated for 18 h with 100 ng/ml lipopolysaccharide (LPS; Sigma-Aldrich) to assess their gene expression. Medium was stored for protein measurement.

### T Cell Proliferation

Spleen and inguinal lymph nodes (LN) were isolated from mice 9 days after EAE induction. T cells were isolated from LN and spleen and cultured in RPMI medium containing 20 µM β-mercaptoethanol, 2% mouse serum, 1% non-essential amino acids, 1% sodium pyruvate, 0.5% P/S. Cells were plated in a 96-well plate at a density of 3 × 10^5^ cells/well and subsequently stimulated with 10 µg/ml MOG for 48 h. Next, 1μCi [^3^H] thymidine (Amersham biosciences, UK) was added for 18 h after which cells were collected using an automatic cell harvester (Pharmacia, Uppsala, Sweden). A β plate liquid scintillation counter (Perkin Elmer, lifesciences, Wellesly, MA, USA) was used to quantify radioactivity and results are expressed as stimulation index (SI). The SI shows the relative proliferation of MOG-stimulated T cells compared to non-stimulated T cells (SI = 1).

### Quantitative PCR (qPCR)

RNA was extracted from tissue or cells using the RNeasy mini kit (Qiagen, Venlo, The Netherlands). In short, lysis was performed with Qiazol Lysis reagent (Qiagen) supplemented with 1% β-mercaptoethanol (Sigma-Aldrich). RNA concentration and quality were determined with a Nanodrop spectrophotometer (Isogen Life Science, The Netherlands). cDNA synthesis was conducted using the Quanta qScript cDNA synthesis kit (Quanta Biosciences, Boston, MA, USA) per manufacturer’s instructions. qPCR was performed on a StepOnePlus™ Real-Time PCR system (Applied biosystems, Ghent, Belgium) using the SYBR green method (Applied Biosystems). The master mix contained 1× SYBR green, 10 µM primers, 12.5 ng cDNA, and nuclease free water. Data were normalized to the most stable reference genes and the ΔΔCt method was used to determine relative quantification of gene expression. Primers were designed using NCBI’s Primer-blast (details are shown in Table 1).

**Table 1:** Mouse primers for RT-qPCR.

Gene	Sequence (5′–3′)	Product size (bp)	Accession number
apoE forward	ACTGGGTCGCTTTTGGGATT	64	NM_000041.2
apoE reverse	CTCCTCCTGCACCTGCTCA		
Tnfα forward	CCAGACCCTCACACTCAG	79	NM_013693.3
Tnfα reverse	CACTTGGTGGTTTGCTACGAC		
iNOS forward	GGCAGCCTGTGAGACCTTTG	150	NM_010927.3
iNOS reverse	GCATTGGAAGTGAAGCGTTTC		
IL-6 forward	TGTCTATACCACTTCACAAGTCGGAG	79	NM_001314054.1
IL-6 reverse	GCACAACTCTTTTCTCATTTCCAC		
Ywhaz forward	GCAACGATGTACTGTCTCTTTTGG	149	NM_001253807.1
Ywhaz reverse	GTCCACAATTCCTTTCTTGTCATC		
Pgk1 forward	GAAGGGAAGGGAAAAGATGC	138	NM_008828.3
Pgk1 reverse	GCTATGGGCTCGGTGTGC		
Rpl13a forward	GGATCCCTCCACCCTATGACA	131	NM_009438.5
Rpl13a reverse	CTGGTACTTCCACCCGACCTC		
Ywhaz forward	GCAACGATGTACTGTCTCTTTTGG	149	NM_001253807.1
Ywhaz reverse	GTCCACAATTCCTTTCTTGTCATC		
T-bet forward	CCATGTGACCCAGATGATCG	87	NM_019507.2
T-bet reverse	TCTGGCTCTCCATCATTCAC		
GATA3 forward	CTGCGGACTCTACCATAA	114	NM_001355111.1
GATA3 reverse	GTGGTGGTCTGACAGTTC		
RORγt forward	GGATGAGATTGCCCTCTA	141	NM_011281.3
RORγt reverse	CCTTGTCGATGAGTCTTG		
Foxp3 forward	CCCAGGAAAGACAGCAACCTT	133	NM_001199347.1
Foxp3 reverse	CTGCTTGGCAGTGCTTGAGAA		
IL-12 forward	TCATCAGGGACATCATCAAACC	210	NM_001303244.1
IL-12 reverse	CGAGGAACGCACCTTTCTG		
Arg1 forward	GTGAAGAACCCACGGTCTGT	132	NM_007482.3
Arg1 reverse	GCCAGAGATGCTTCCAACTG		
IL-17 forward	ATCAGGACGCGCAAACATGA	125	NM_010552.3
IL-17 reverse	TTGGACACGCTGAGCTTTGA		
IL-4 forward	CTCACAGCAACGAAGAACACCA	148	NM_021283.2
IL-4 reverse	AAGCCCGAAAGAGTCTCTGCA		
IFNγ forward	TGAGGTCAACAACCCACAGGT	104	NM_008337.4
IFNγ reverse	GACTCCTTTTCCGCTTCCTGAG		

### NO Production

Peritoneal macrophages in culture were stimulated with 100 ng/ml LPS (Sigma-Aldrich) prior to the assay. NO production was determined in the supernatant of peritoneal macrophages using a Griess-reagent assay (Promega, Leuven, Belgium) according to the manufacturer’s instructions.

### Immunohistochemistry

Animals were perfused transcardially through the ventricular catheter with ringer (containing heparin). Spinal cord tissue was isolated, snap-frozen, and sectioned with a Leica CM1900UV cryostat (Leica Microsystems, Wetzlar, Germany) to obtain 8 µm slices. Dried cryosections were fixed in acetone for 10 min and subsequently blocked for 0.5 h with 10% normal serum. Cryosections were incubated overnight at 4°C with primary antibodies. Next, secondary antibodies were added for 1 h. Primary antibodies used were rat anti-mouse CD3 (1:150; AbD Serotec) and rat anti-mouse F4/80 (1:100; AbD Serotec). Goat anti-rat Alexa Fluor^®^555 (1:400; Invitrogen) was used as a secondary antibody. Nuclei were stained with 4′,6-diamidino-2-phenylindole (DAPI; 1:2,500; Sigma-Aldrich). Stained sections were visualized under a Nikon Eclipse 80i fluorescence microscope (Nikon, Kingston, UK). Sections were analyzed using NIS Elements viewer software 4.0 (Nikon).

### Western Blot

Apolipoprotein E secretion by peritoneal macrophages was determined using western blot. Culture medium was collected and separated *via* sodium dodecyl sulfate polyacrylamide gel electrophoresis (SDS-PAGE). Gels were transferred to a PVDF-membrane (VWR, Leuven, Belgium) and blots were blocked for 1 h in TBS-Tween 5% non-fat dry milk. Membranes were probed with mouse anti-apoE (Abbiotec, Antwerpen, Belgium). After washing steps with TBS-Tween, blots were incubated with horseradish peroxidase-labeled anti-mouse antibody (Dako, Heverlee, Belgium). Immunoreactive signals were detected with Enhanced Chemiluminescence (ECL Plus, GE Healthcare, Diegem, Belgium).

### Phagocytosis Assay

Myelin was isolated from brain tissue of healthy adult WT C57BL/6 OlaHSD mice (Harlan) by means of sucrose density gradient centrifugation, as previously described (31). Next, myelin was labeled with the lypophilic dye 1,1′-Dioctadecyl-3,3,3′,3′-Tetramethylindocarbocyanine Perchlorate (DiI) (Thermofisher Scientific, Erembodegem, Belgium). Peritoneal macrophages were incubated with DiI labeled myelin (25 µg/ml) for 90 min at 37°C and 5% CO_2_. Next, cells were rinsed with PBS (Sigma-Aldrich), detached with PBS/EDTA and resuspended in FACS buffer containing 1× PBS, 2% FCS (Hyclone) and sodium Azide. The fluorescent internalized myelin was measured using the FACS Calibur flow cytometer (BD biosciences, Erembodegem, Belgium). Results are expressed as mean fluorescence.

### ELISA

Peritoneal mouse macrophages were stimulated with 100 ng/ml LPS for 18 h prior to the assay. TNFα concentration in peritoneal macrophage culture supernatant was determined using the TNFα Mouse Uncoated ELISA Kit (Thermofisher), following the manufacturer’s instructions. Absorption was measured at 450 nm using a microtiterplate reader (Biorad, Temse, Belgium).

### Statistical Analysis

Data were statistically analyzed using GraphPad Prism for windows (version 5.0) and are reported as mean ± SEM. D’Agostino and Pearson omnibus normality test was used to test Gaussian distribution. A two-tailed unpaired student *t*-test was used for normally distributed data sets [*t*(df); P]. The Mann–Whitney analysis (MWU, n1, n2; P) was used for data sets that did not pass normality. EAE scores were analyzed using two-way ANOVA (Bonferroni’s *post hoc* multiple comparison test). *p < 0.05, **p < 0.01, and ***p < 0.001.

## Results

### *Ldlr* Deficiency Reduces EAE Severity in Female But Not in Male Mice

**Figure 1:**
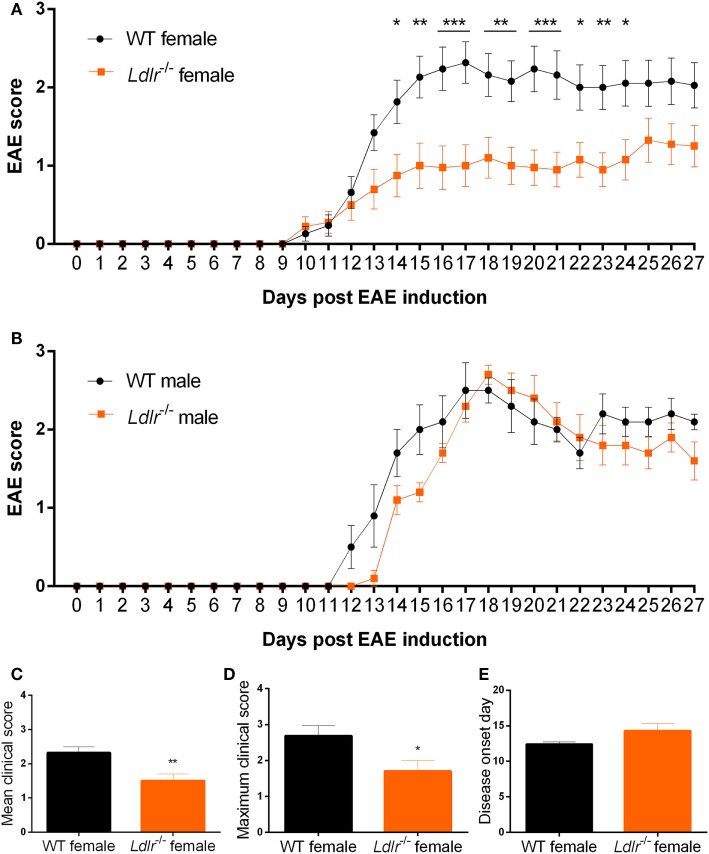
*Ldlr*^−/−^ reduces experimental autoimmune encephalomyelitis (EAE) severity in female but not in male mice. Daily EAE score of female [*p* < 0.0079; wild-type (WT) vs *ldlr*^−/−^ by two-way ANOVA] (*n* = 19–20, pooled from three independent experiments) **(A)** and male (*p* < 0.2221; WT vs *ldlr*^−/−^ by two-way ANOVA) (*n* = 5) **(B)** mice. The mean score [*t*(31) = 3.050; *p* < 0.0048] **(C)**, the maximum score [*t*(37) = 2.349; *p* < 0.0244] **(D)**, and the day of onset of the disease [*t*(29) = 1.848; *p* < 0.0748] **(E)**.

To elucidate whether the LDLr contributes to neuroinflammation, we induced EAE in male and female *ldlr*^−/−^ mice. We show that *ldlr* deficiency has a sex-specific effect on the EAE course. Female *ldlr*^−/−^ mice exhibited a decreased EAE disease severity compared to WT mice, whereas in male mice *ldlr* deficiency did not affect EAE disease severity (Figures 1A,B). In female animals, the mean peak of disease symptoms was reached around day 17. The mean disease score was attenuated in *ldlr*^−/−^ (1.504 ± 0.1995) compared to WT (2.318 ± 0.1782) mice (Figure 1C). Accordingly, the maximum disease score was lower in LDLr^−/−^ (1.70 ± 0.30) compared to WT (2.68 ± 0.29) mice (Figure 1D). The *ldlr*^−/−^ and WT groups displayed a similar day of onset (14.29 ± 1.04 and 12.41 ± 0.34, respectively Figure 1E).

### *Ldlr* Deficiency Has No Significant Influence on Immune Cell Infiltration into the CNS

**Figure 2:**
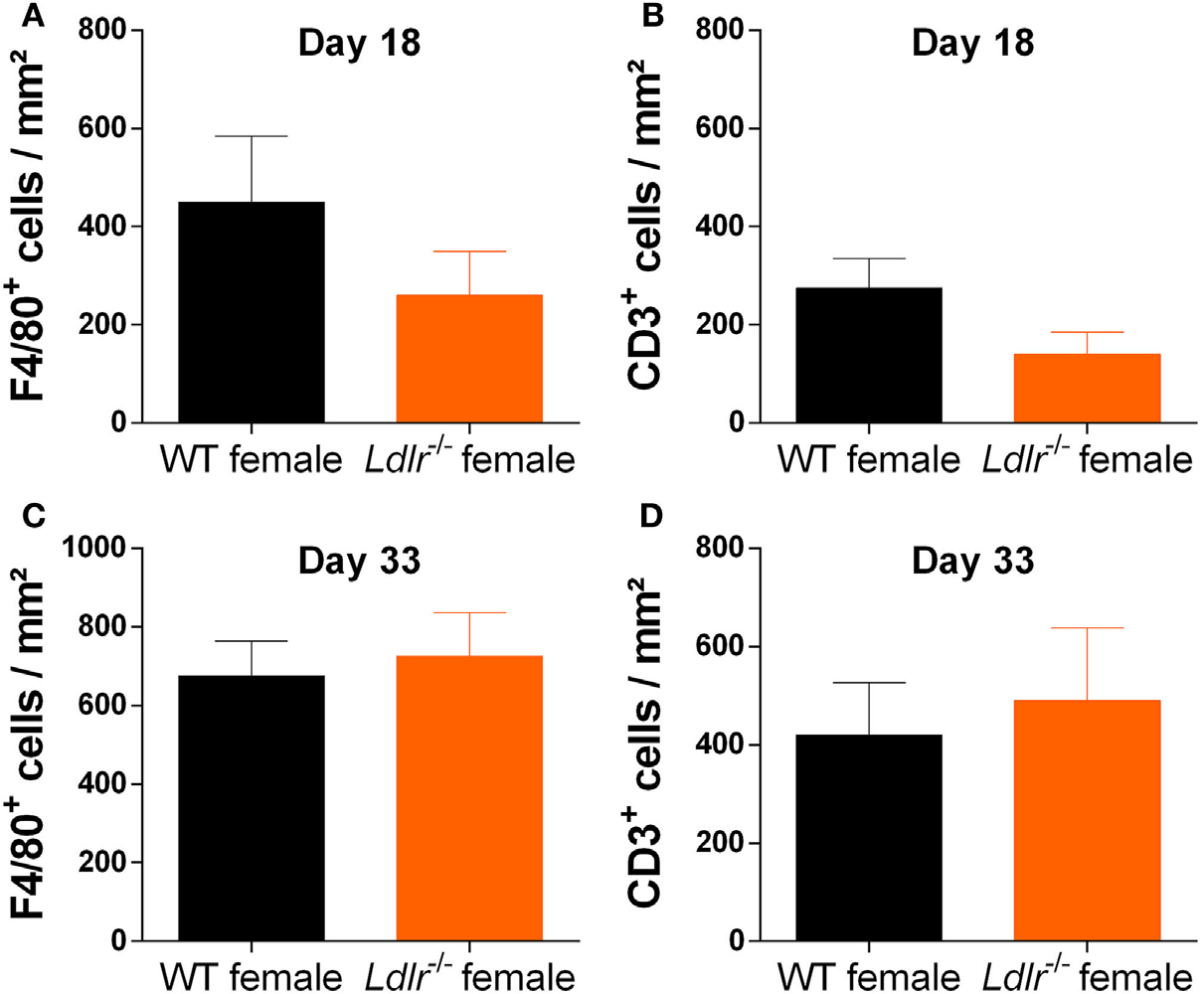
*Ldlr* deficiency has no significant influence on macrophage and T cell infiltration into the central nervous system. Immunohistological sections of spinal cord tissue from wild-type (WT) and *ldlr*^−/−^ mice at day 18 **(A,B)** and day 33 **(C,D)** post immunization. Sections were stained against F4/80 for macrophages **(A,C)** and CD3 for T cells **(B,D)**. At least six sections per mouse were quantified (*n* = 5). Representative images can be found in the supplementary material section S1/2.

Experimental autoimmune encephalomyelitis is characterized by the infiltration of peripheral immune cells into the CNS leading to a local inflammatory response. To determine whether this process is altered by *ldlr* deficiency in female mice, the accumulation of T cells (CD3) and macrophages (F4/80) in the CNS of WT and *ldlr* deficient female EAE mice was assessed by immunohistochemistry at day 18 and day 33 post immunization (Figure 2; Figures S1 and S2 in Supplementary Material). Despite a reduced disease severity, no significant differences in the number of infiltrated macrophages and T cells into the spinal cord tissue were observed comparing female WT EAE mice and *ldlr*-deficient EAE mice, although a trend on day 18 is visible.

### *Ldlr* Deficiency in Female Mice Has No Influence on T Cell Proliferation

**Figure 3:**
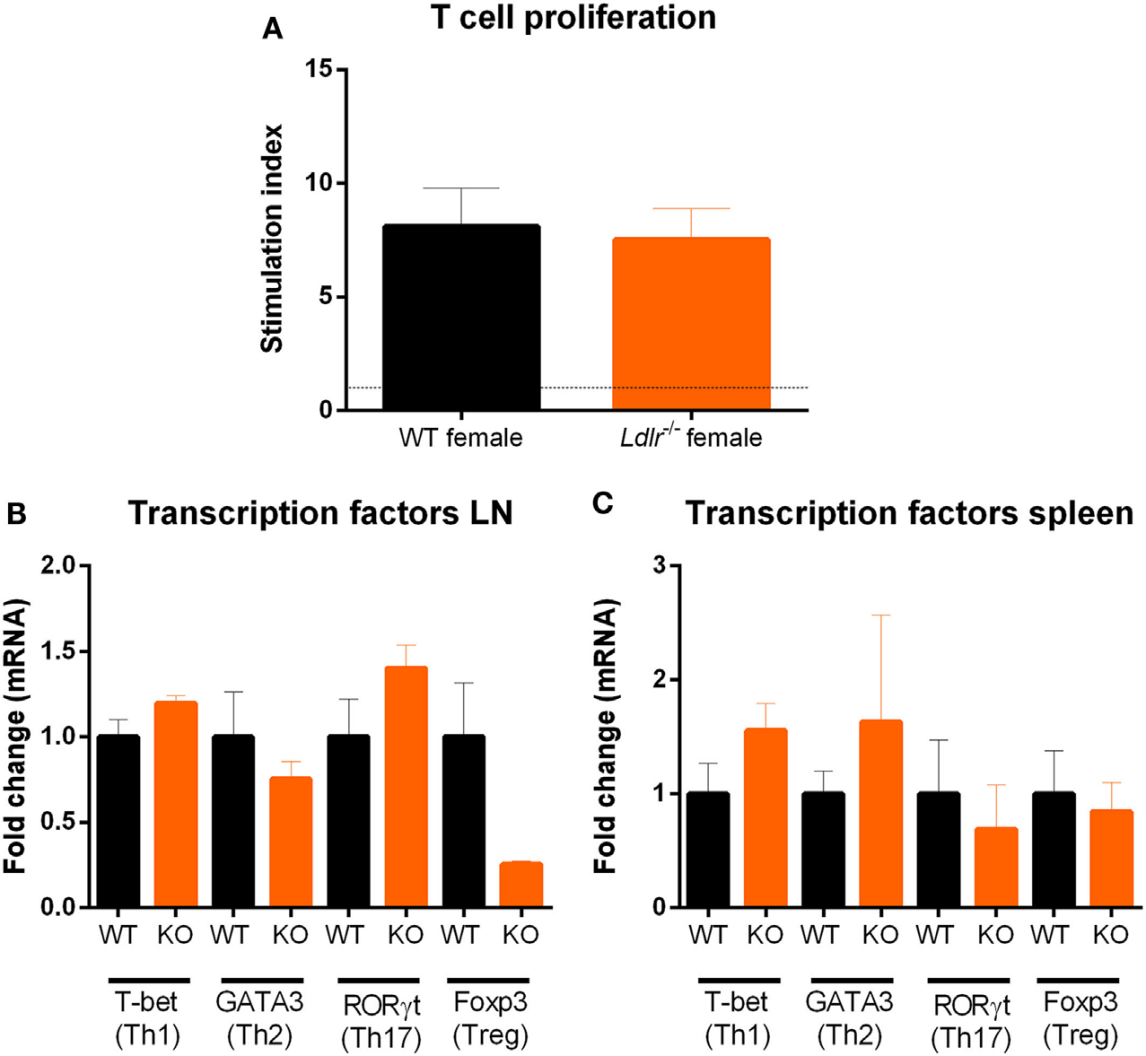
*Ldlr* deficiency has no influence on T cell proliferation. Stimulation index (SI) of T cells isolated from lymph nodes (LN) of wild-type (WT) and LDLR deficient mice (*n* = 3). Cells were collected on day 9 post immunization and proliferation was analyzed by means of a thymidine assay. Unstimulated cells an SI of 1 **(A)**. Gene expression of transcription factors (T-bet, GATA3, RORγt, and Foxp3) in T cells isolated from LN **(B)** and spleen **(C)** from WT and *ldlr*^−/−^ female mice on day 9 post immunization (*n* = 4).

T cell proliferation is an important hallmark of EAE and is essential for the initiation of EAE pathogenesis. Since T cells are dependent on cholesterol in order to proliferate (32), we investigated the influence of *ldlr* on T cell proliferation during EAE. T cells from both *ldlr*^−/−^ and WT mice were isolated on day 9 post immunization and a thymidine assay was used to determine their proliferation capacity. No significant differences in T cell proliferation between *ldlr*^−/−^ and WT mice induced with EAE (Figure 3A). In addition, we determined mRNA expression of transcription factors such as T-bet, GATA3, RORγt, and Foxp3, which are involved in T cell differentiation into Th1, Th2, Th17, and Treg subsets, in LN and spleen but found no difference between *ldlr*^−/−^ and WT female mice (Figures 3B,C). Lastly, we analyzed the mRNA expression levels of cytokines important in T cell differentiation (IFNγ, IL-4, IL-17, and IL-10) in T cells isolated from both spleen and LN. No differences in the gene expression of these cytokines were observed. The mRNA levels of IL-4 and IL-17 were not detectable. Data are shown in the supplementary section (Figure S3 in Supplementary Material). Together, these results indicate that altered T cell proliferation and differentiation do not account for the reduced EAE in *ldlr* deficient mice.

### *Ldlr* Deficiency Reduces Inflammation in the Spinal Cord of Female Mice Compared to Male Mice

**Figure 4:**
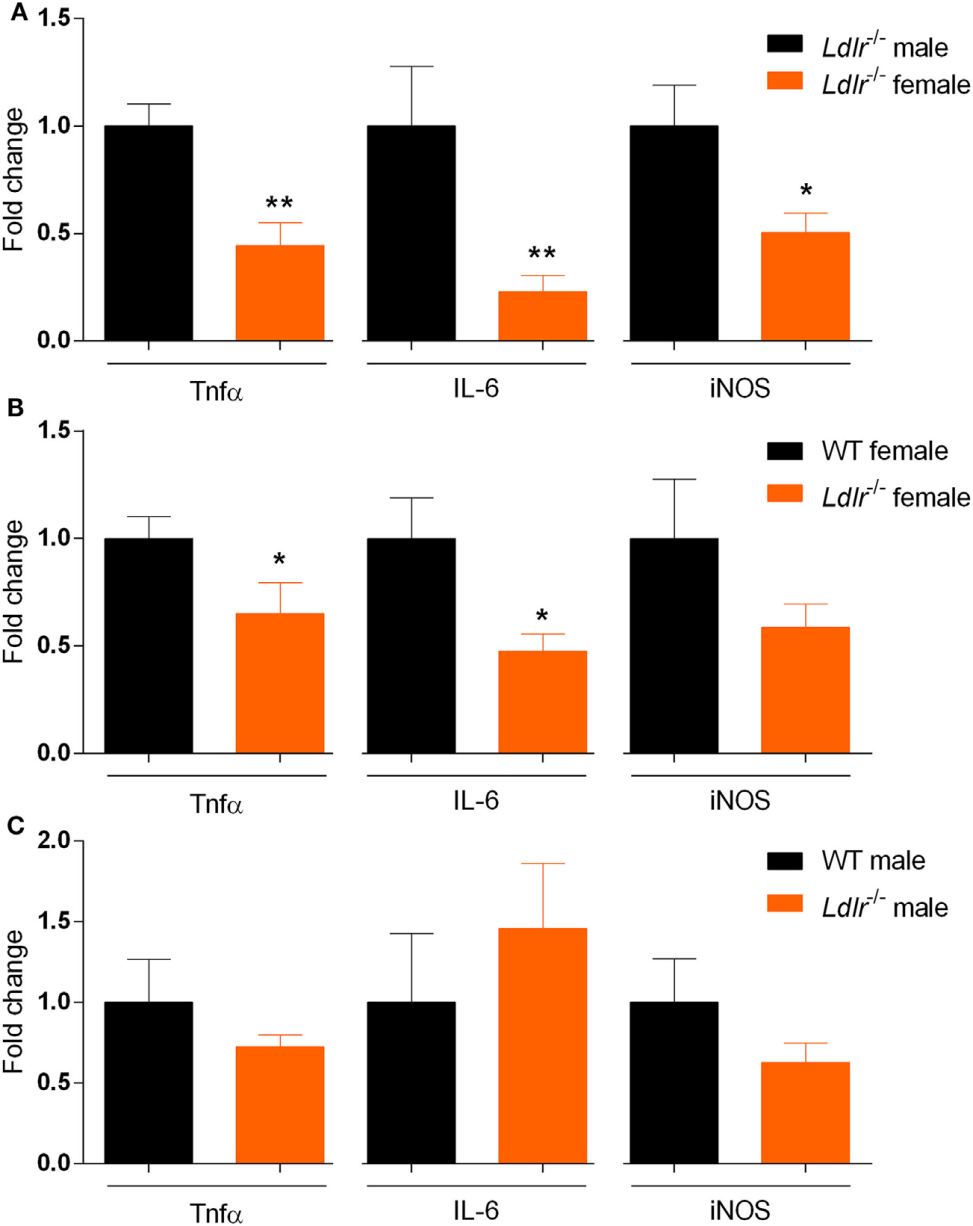
Relative expression of proinflammatory genes in the spinal cord of *ldlr*^−/−^ mice. Expression of *Tnf*α [*t*(10) = 3.668; *p* < 0.0044], *IL-6* (MWU = 0; 5; 7; *p* < 0.0026) and *iNOS* [*t*(10) = 2.588; *p* < 0.0271] for male and female *ldlr*^−/−^ mice at experimental autoimmune encephalomyelitis (EAE) peak (*n* = 5–7) **(A)**. Expression of *Tnf*α [*t*(13) = 2.467; *p* < 0.0284], *IL-6* [*t*(12) = 2.553; *p* < 0.0254], and *iNOS* [*t*(13) = 1.313; *p* < 0.2118] for wild-type (WT) and *ldlr*^−/−^ female mice at EAE peak (*n* = 5–7) **(B)**. Expression of *Tnf*α [*t*(7) = 1.106; *p* < 0.3054], *IL-6* [*t*(7) = 0.7745; *p* < 0.4641], and *iNOS* [*t*(7) = 1.370; *p* < 0.2129] for WT and *ldlr*^−/−^ male mice at EAE peak (*n* = 5–7) **(C)**. Data were normalized to the most stable reference genes, determined by Genorm (*Ywhaz* and *Pgk1*).

Our data indicate that *ldlr* deficiency in female mice attenuates EAE severity without affecting T cell proliferation and the level of immune cell infiltration into the CNS. To further define the inflammatory burden in the CNS, whole spinal cord tissue of male and female *ldlr*^−/−^ mice was analyzed for mRNA expression of proinflammatory genes. Female *ldlr*^−/−^ mice exhibited a significantly lower expression of tumor necrosis factor alpha (*Tnf*α), Interleukin-6 (*IL-6*), and nitric oxide synthase (*iNOS*) compared to male *ldlr*^−/−^ mice and female WT mice (Figures 4A,B), whereas the expression of these inflammatory genes is not changed in male *ldlr*^−/−^ mice (Figure 4C). These data indicate that female *ldlr*^−/−^ mice have decreased inflammation in the spinal cord compared to male *ldlr*^−/−^ and female WT mice.

### *Ldlr* Deficiency Suppresses the Inflammatory Phenotype of Macrophages in Female Mice

**Figure 5:**
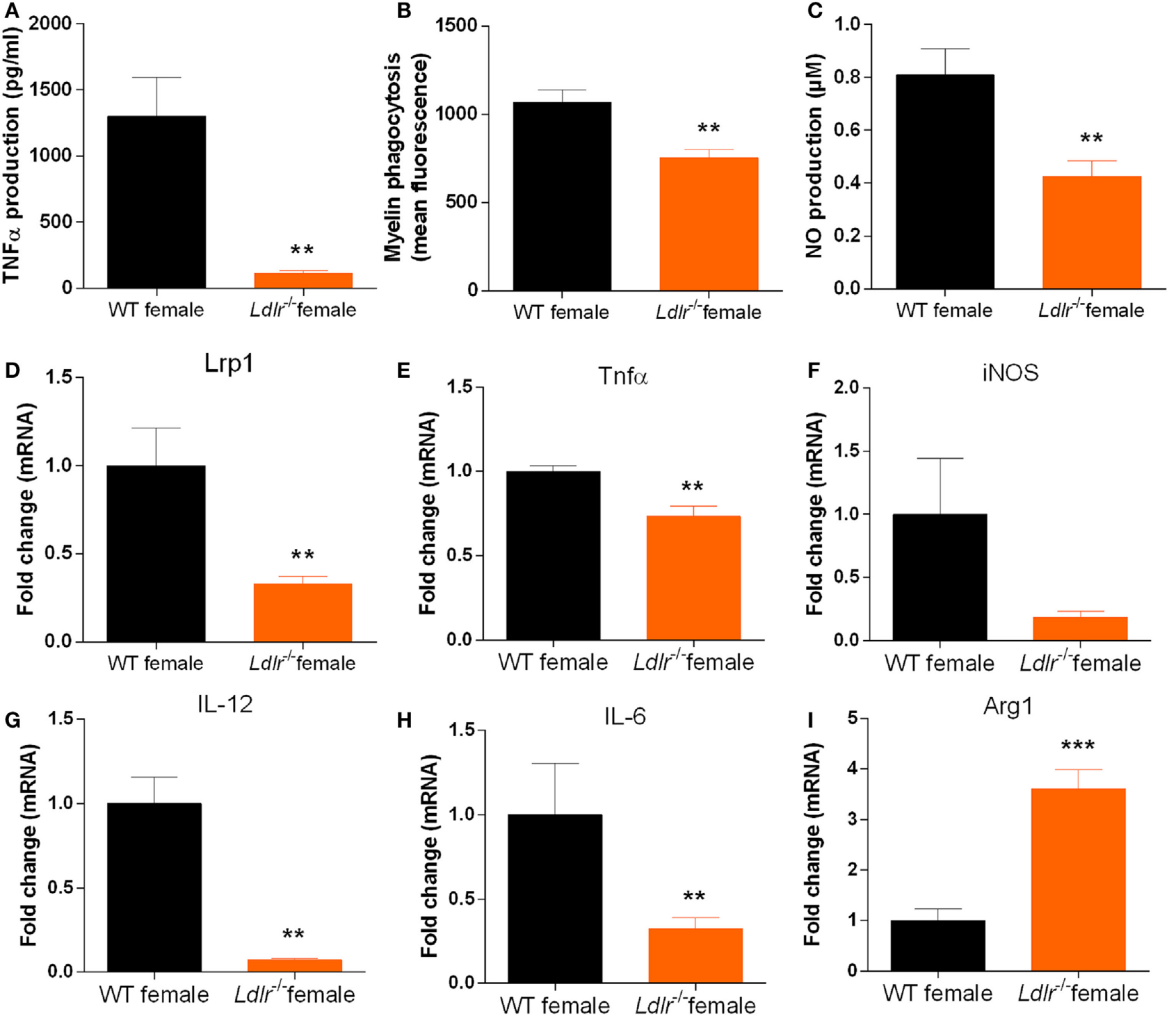
*Ldlr* deficiency influences the production of inflammatory mediators by macrophages. TNFα production [*t*(17) = 3.444; *p* < 0.0032] (*n* = 6) **(A)**, NO production [*t*(22) = 3.368; *p* < 0.0029] (*n* = 6) **(B)**, phagocytosis (mean fluorescence) [*t*(28) = 3.638; *p* < 0.0012] (*n* = 6) **(C)**, *lrp1* expression (MWU = 2; 5; 8; *p* < 0.0063) (*n* = 5–8) **(D)**, *Tnf*α expression [*t*(9) = 3.630; *p* < 0.0055] **(E)**, i*NOS* expression [*t*(9) = 2.020; *p* < 0.0742] **(F)**, *IL-12* expression (MWU = 0; 4; 6; *p* < 0.0095) **(G)**, *IL-6* expression [*t*(9) = 2.391; *p* < 0.0405] **(H)**, and *Arg1* expression [*t*(8) = 5.846; *p* < 0.0004] **(I)** in peritoneal macrophages isolated from wild-type (WT) and *ldlr*^−/−^ female mice (*n* = 6).

As the *ldlr* is highly expressed on macrophages and we observed a reduced expression of cytokines typically released by macrophages in female *ldlr*^−/−^ EAE mice, we investigated the influence of *ldlr* deficiency on the inflammatory phenotype of macrophages *in vitro*. We found that female LPS-stimulated *ldlr*^−/−^ macrophages produce less TNFα and NO compared to macrophages isolated from WT females (Figures 5A,B). In addition, we show that macrophages isolated from *ldlr*^−/−^ female mice less efficiently internalize myelin compared to macrophages isolated from WT females (Figure 5C). Interestingly, expression of *lrp1*, a major receptor involved in the phagocytosis of myelin (33), was reduced as well (Figure 5D). The expression of the proinflammatory genes *Tnf*α, *IL-6 and Il-12* is reduced in *ldlr*^−/−^ female mice, whereas the anti-inflammatory gene *Arg1* is increased in macrophages isolated from *ldlr*^−/−^ female mice compared to WT mice (Figures 5E–I). These data indicate that macrophages isolated from female *ldlr*^−/−^ mice have an attenuated inflammatory response upon stimulation and have a reduced phagocytosis capacity compared to their WT counterparts.

### ApoE Expression Is Increased in Activated Peritoneal Macrophages Isolated from *ldlr*^−/−^ Female Mice

**Figure 6:**
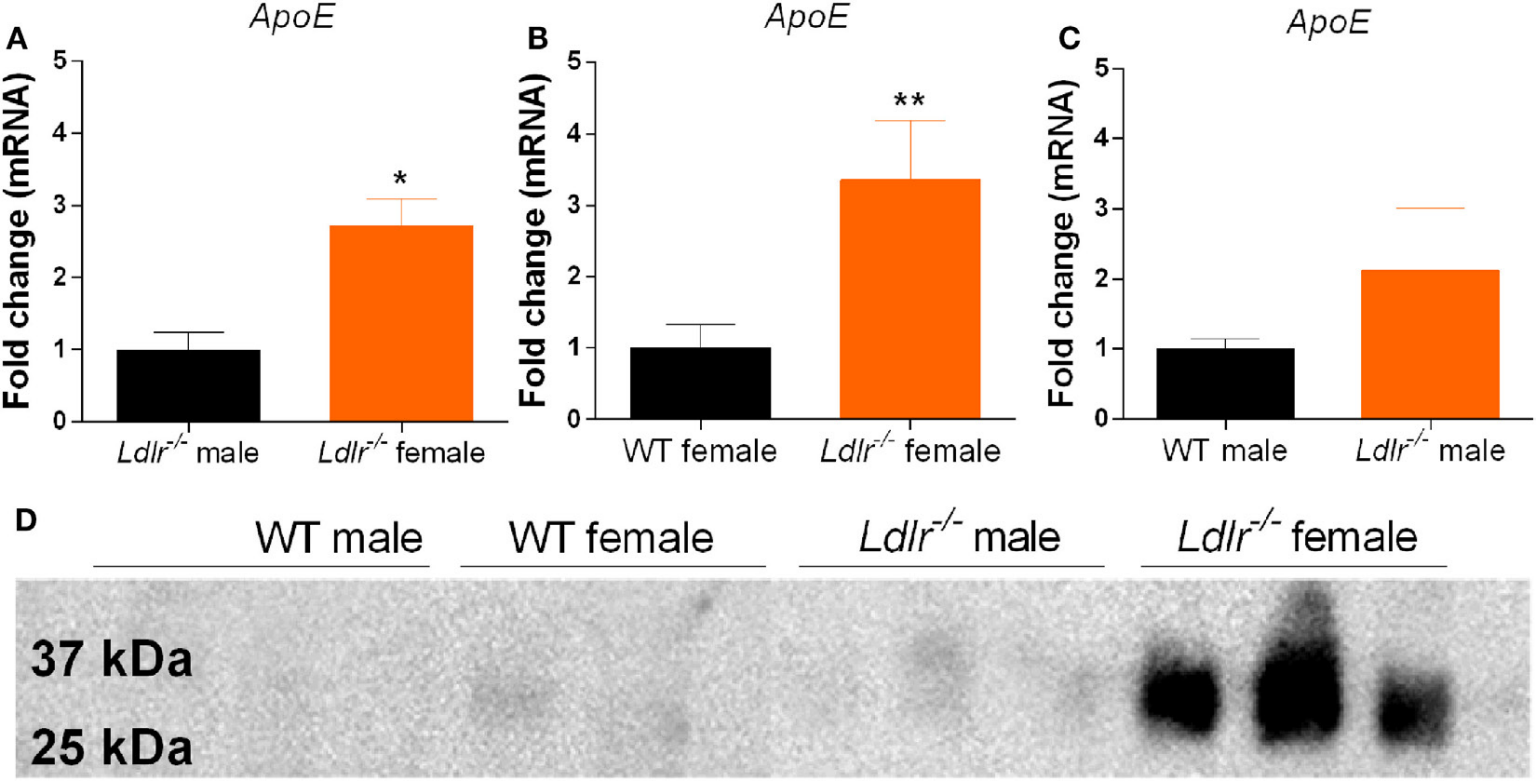
*Apolipoprotein E* (*ApoE*) expression by peritoneal macrophages. Comparison of *apoE* gene expression between male and female *ldlr*^−/−^ mice (MWU = 1; 6; 6; *p* < 0.0476) (*n* = 6) **(A)**, wild-type (WT) and *ldlr*^−/−^ female mice (MWU = 1; 6; 6; *p* < 0.0044) (*n* = 6) **(B)**, and WT and *ldlr*^−/−^ male mice (*n* = 6) **(C)**. Western blot analysis of cell culture medium from LPS-stimulated peritoneal macrophages, stained for apoE (*n* = 3) **(D)**.

Apolipoprotein E is known to induce an anti-inflammatory phenotype in macrophages (34). Interestingly, we show that ApoE mRNA expression is significantly increased in activated peritoneal macrophages from *ldlr*^−/−^ female mice compared to both male *ldlr*^−/−^ mice and female WT mice (Figures 6A–C). Next, we used Western blot to determine the amount of apoE secreted into the culture medium. We found that only LPS-stimulated macrophages isolated from female *ldlr*^−/−^ mice, and not those from WT and male *ldlr*^−/−^ mice, secreted significant levels of apoE (Figure 6D). These data suggest that *ldlr* deficiency in female mice is responsible for the increase and subsequent secretion of apoE by macrophages.

### Lack of apoE Abrogates the Reduced Neuroinflammatory Response in Female *ldlr*^−*/*−^ Mice

**Figure 7:**
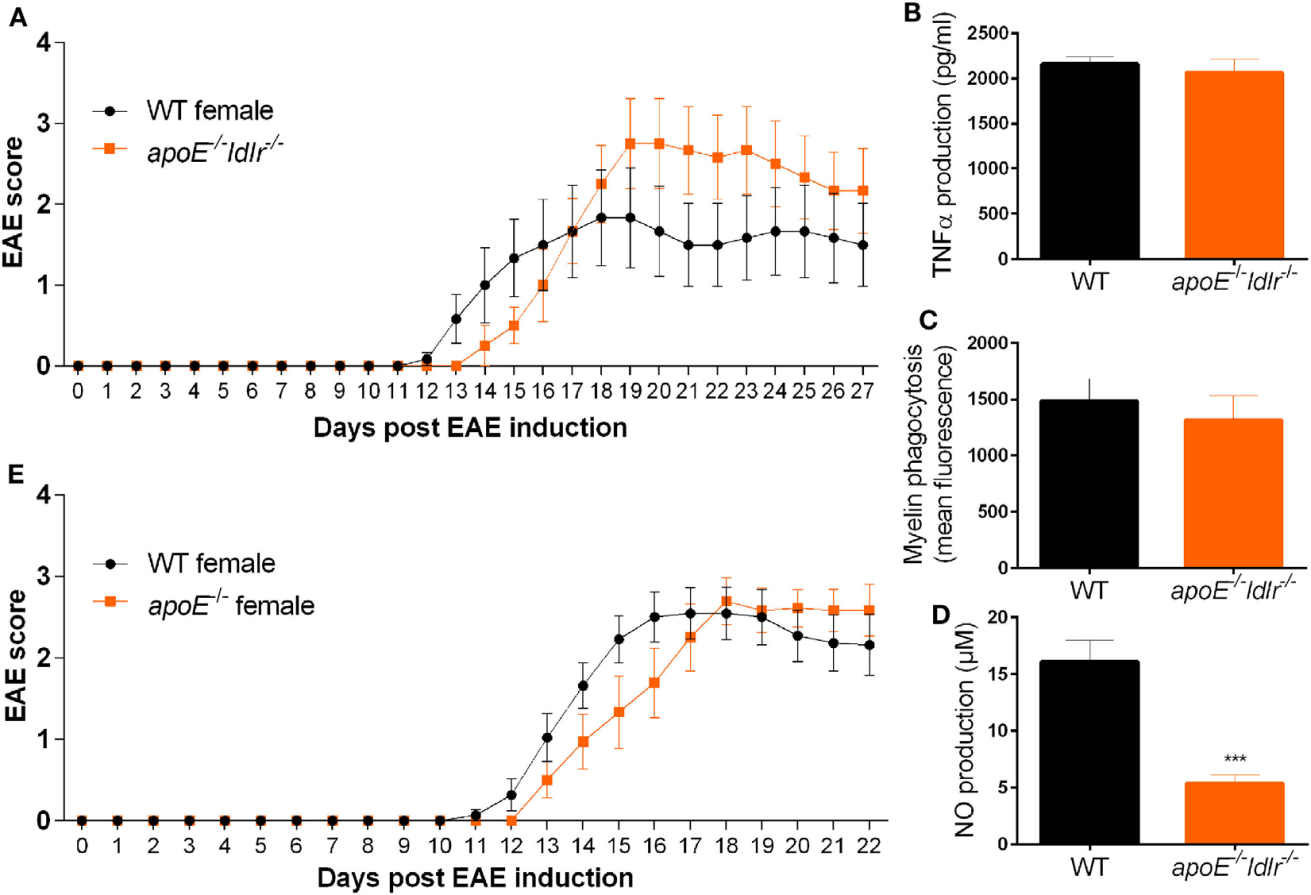
Lack of apolipoprotein E (apoE) abrogates the reduced neuroinflammatory response in female *ldlr*^−^*^/^*^−^ mice. Daily clinical EAE score of female WT and *apoE*^−/−^ and *ldlr*^−/−^ mice (*n* = 6) **(A)**. ELISA to measure TNFα production (*n* = 6) **(B)**, myelin phagocytosis assay (*n* = 6) **(C)**, Griess assay to measure NO production [*t*(15) = 5.091; *p* < 0.0001] (*n* = 6) **(D)**, for WT and *apoE*^−/−^ and *ldlr*^−/−^ female mice. *ApoE*^−/−^ female mice show a similar EAE disease score compared to WT female mice (*n* = 9–11) **(E)**.

To define to what extend an increase in apoE accounts for the observed reduction in EAE disease severity in *ldlr*^−/−^ female mice and the observed less-inflammatory phenotype of macrophages isolated from these mice, we crossbred *apoE*^−/−^ *and ldlr*^−/−^ mice. In contrast to female *ldlr*^−/−^ mice, female *apoE*^−/−^*ldlr*^−/−^ mice did not exhibit an attenuated EAE disease course compared to WT mice (Figure 7A). In addition, the observed decreased capacity to phagocytose myelin and produce TNFα by macrophages from *ldlr*^−^*^/^*^−^ female mice was counteracted by depletion of apoE (Figures 7B,C). Interestingly, the impairment in NO production observed in *ldlr*^−/−^ macrophages was also present in *apoE*^−/−^*ldlr*^−/−^ macrophages indicating apoE is not accountable for this effect (Figure 7D). *ApoE*^−/−^ alone did not alter EAE severity in female mice compared to WT mice (Figure 7E). These data indicate that the lack of apoE largely restores the impaired inflammatory response induced by *ldlr* deficiency in female mice and that the effect of *ldlr*^−/−^ on NO production is not responsible for the ameliorated disease course in these mice.

## Discussion

In this study, we show that *ldlr*^−/−^ attenuates neuroinflammation in female but not in male EAE mice, and that macrophages isolated from female mice have an attenuated inflammatory response and reduced phagocytic capacity. Interestingly, apoE deficiency counteracts the impact of *ldlr* deficiency in females on the course and pathology of EAE. These findings indicate that an increased apoE expression in macrophages apoE in female *ldlr*^−/−^ mice is at least partially responsible for the observed reduction in EAE severity.

We show that *ldlr* deficiency in female mice decreases the inflammatory burden in the CNS but does not affect T cell proliferation and differentiation, and immune cell infiltration into the CNS. However, the levels of proinflammatory mediators in the CNS were reduced in these mice and isolated macrophages showed an impaired inflammatory response. These findings suggest that *ldlr* deficiency affects the inflammatory and phagocytic properties of macrophages, thereby altering EAE disease severity. In concordance, levels of the proinflammatory markers *Tnf*α, *IL-6, and Il-12* were reduced while an increased gene expression of the anti-inflammatory marker *Arg1 was observed* in macrophages isolated from *ldlr*^−/−^ female mice compared to WT macrophages. Moreover, *ldlr* deficiency decreases macrophage mediated myelin phagocytosis and expression of *lrp1*. LRP1 is a member of the LDLr family that also functions as a scavenger receptor and has been shown to be directly involved in myelin phagocytosis (33). Myelin degradation and phagocytosis by macrophages enhances demyelination which leads to neurological symptoms in MS (3). Although speculative, it is possible that lack of *ldlr* results in decreased *Lrp1* expression, thereby indirectly impairing myelin phagocytosis. Together, an altered macrophage response at least partially accounts for the beneficial effects of *ldlr* deficiency on EAE severity.

In contrast to the reduction in EAE severity in *ldlr*^−/−^ mice, the clinical outcome was not altered in *apoE*^−/−^*ldlr*^−/−^ mice. Activated peritoneal macrophages isolated from *ldlr*^−/−^ female mice significantly increased *apoE* expression compared to WT or their male counterparts and, the beneficial effect of *ldlr* deficiency on myelin phagocytosis and TNFα production was absent in *apoE*^−/−^*ldlr*^−/−^ macrophages. Although the LDLr is described to directly regulate apoE levels in the CNS (35), we found no elevated apoE gene expression in the CNS of *ldlr*^−/−^ EAE mice (data not shown). Studies have reported that increased expression of apoE leads to a less-inflammatory phenotype in macrophages by downregulating M1-like and upregulating M2-like markers. In addition, apoE suppresses microglial activation and the release of TNFα (34, 36, 37). Furthermore, treatment of EAE animals with apoE-derived peptides ameliorates the clinical course of EAE and apoE deficiency exacerbates EAE by increasing immune reactivity and hampering CNS repair mechanisms (38, 39). These findings suggest that the LDLr suppresses ApoE expression thereby exacerbating neuroinflammatory responses in females.

The reason for the sexual dimorphism in apoE expression remains elusive. It is reported that apoE exerts moderate sex-specific effects on neuroinflammation. Schrewe and coworkers found that the EAE disease course was slightly attenuated in male *apoE*^−/−^ mice, whereas EAE was more severe in female *apoE*^−/−^ mice compared to WT mice. In our hands *apoE*^−/−^ did not alter EAE disease outcome in female mice compared to WT mice. Methodological differences (e.g., immunization protocol) may explain these discrepancies. Moreover, in Schrewe’s study differences between WT and *apoE*^−^*^/^*^−^ female mice were only visible after 30–36 days post immunization, whereas our experiment lasted for up to 22 days post immunization. Nevertheless, the question remains why only macrophages from *ldlr*^−/−^ female mice abundantly secrete apoE. Macrophages express receptors for sex hormones such as estrogen and testosterone and these hormones modulate macrophage activity (40, 41). Interestingly, administration of estrogens has been shown to directly regulate hepatic LDLr activity in human cells *in vitro* and 17β-estradiol increases the expression of apoE during the maturation stage of human monocytes to macrophages (42–44). As lack of LDLr has been shown to increase apoE levels also (35), both mechanisms may contribute to the increased apoE levels observed in female *ldlr*^−^*^/^*^−^ mice.

However, it remains to be elucidated whether sex hormones are exclusively responsible for the observed effects. Studies investigating plasma lipid and lipoprotein concentrations across the menstrual cycle in women report virtually no variation (45–47), and those that do, report only small reductions in LDL during the luteal phase and no changes in plasma triglyceride or HDL concentrations (45, 46). Therefore, the sexual dimorphism in lipid metabolism and its impact on neuroinflammation appears to be the result of an intricate network of sex hormones in combination with other modulators of lipid metabolism. The key pathways and mediators of this network remain to be elucidated, but there are many other factors involved such as insulin and adipokines (48), and gene expression and imprinting (49), that need to be explored in the future.

In addition to apoE, differences in female and male mice may be the result of differences in LDLr levels and function. However, there are few studies about sex-related differences in LDLr behavior. Segatto and coworkers determined age- and sex-related differences in extra-hepatic LDLr expression and found that LDLr protein expression was decreased in 12-month-old (onset of menopause) female rat brains, which was completely restored by estrogen treatment. In skeletal muscle, LDLr levels were increased in both male and female aged rats, whereas in the heart no modifications were observed either in aged rats or rats of a specific gender (50). These data illustrate the complex age- and sex-related and tissue-specific regulation of LDLr expression, and warrant the need of more research to clarify the implications of these differences for neuroinflammatory diseases and other conditions involving the LDLr.

Collectively, we show that *ldlr*^−/−^ attenuates EAE disease severity in female but not in male EAE mice. Although the nature of the observed sexual dimorphism remains unclear, our findings show that LDLr and associated apoE levels are involved in neuroinflammatory diseases such as multiple sclerosis, which may have implications for future treatment strategies.

## Ethics Statement

Experiments were conducted in according to institutional guidelines and were approved by the ethical committee on animal experiments (ECAE) of Hasselt University.

## Author Contributions

JM, ST, KN, JV, TV, and JB performed the experiments and analyzed the data. JM wrote the manuscript. JM, ST, KN, JV, TV, JB, JH, and JH participated in the design and coordination of the project.

## Conflict of Interest Statement

The authors declare that the research was conducted in the absence of any commercial or financial relationships that could be construed as a potential conflict of interest.

## Supplementary Material

Figure S1 Fluorescent staining of macrophages (F4/80) in experimental autoimmune encephalomyelitis spinal cord. Staining of macrophages in wild-type female **(A,C)** and ldlr^−/−^ female mice **(B,D)** on days 18 and 33, respectively.

Figure S2 Fluorescent staining of T cells (CD3) in experimental autoimmune encephalomyelitis spinal cord. Staining of T cells in wild-type female **(A,C)** and ldlr^−/−^ female mice **(B,D)** on day 18 and 33, respectively.

Figure S3 Cytokine important in T cell differentiation. mRNA expression levels of cytokines important in T cell differentiation (IFNγ, IL-4, IL-17, and IL-10) in T cells isolated from both spleen and lymph nodes from WT and ldlr^−/−^ female mice (*n* = 4). No differences in the gene expression of these cytokines were observed. The mRNA levels of IL-4 and IL-17 were not detectable.
