# Supplementary figures and images for: Low-Density Lipoprotein Receptor Deficiency Attenuates Neuroinflammation through the Induction of Apolipoprotein E
Source: Front Immunol. 2017 Nov 30;8:1701. doi: 10.3389/fimmu.2017.01701 (PMC5727422; doi:10.3389/fimmu.2017.01701)

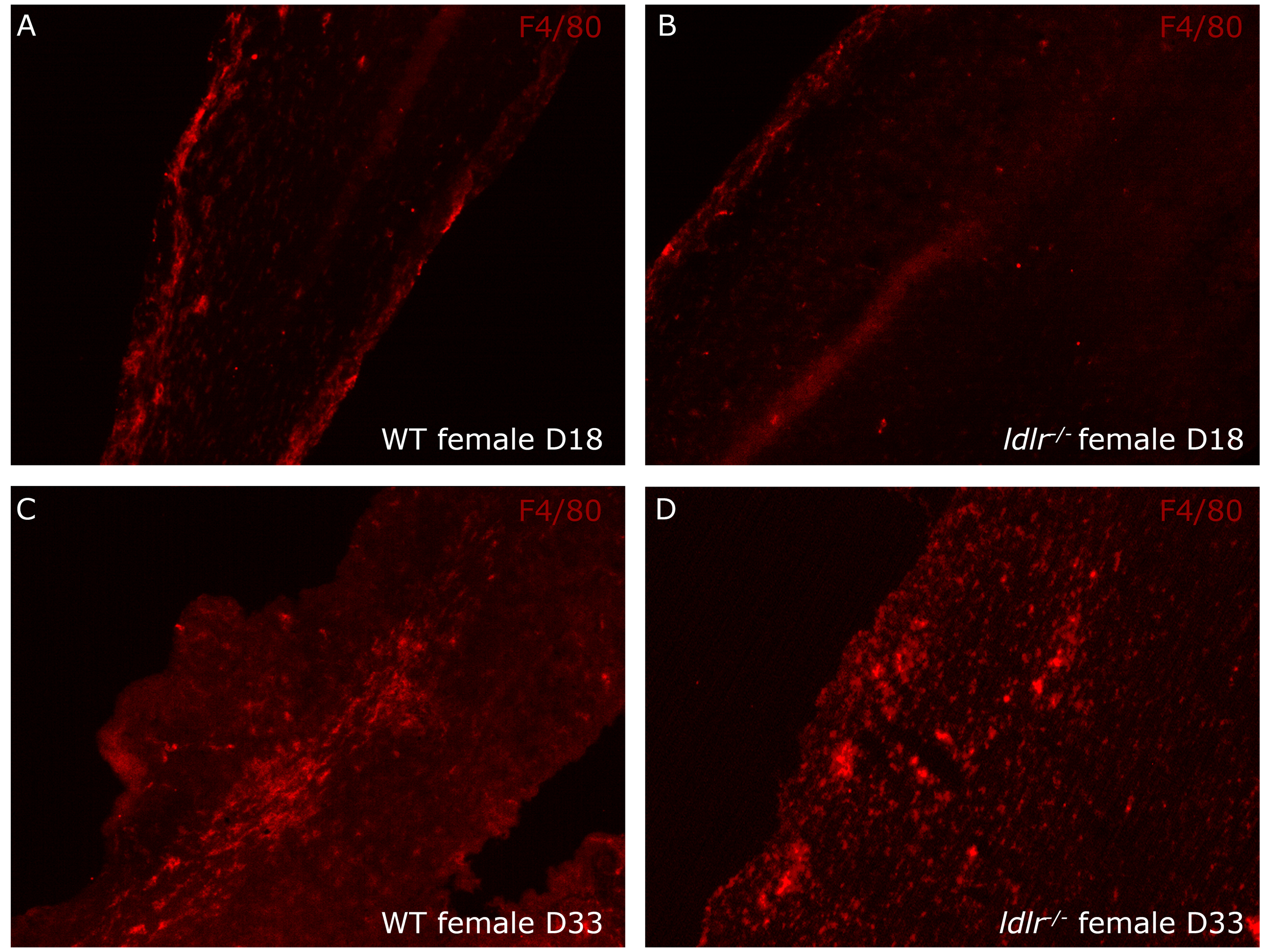

Supplement: Figure S1 — Fluorescent staining of macrophages (F4/80) in experimental autoimmune encephalomyelitis spinal cord. Staining of macrophages in wild-type female (A,C) and ldlr−/− female mice (B,D) on days 18 and 33, respectively. [file Image_1.tif]

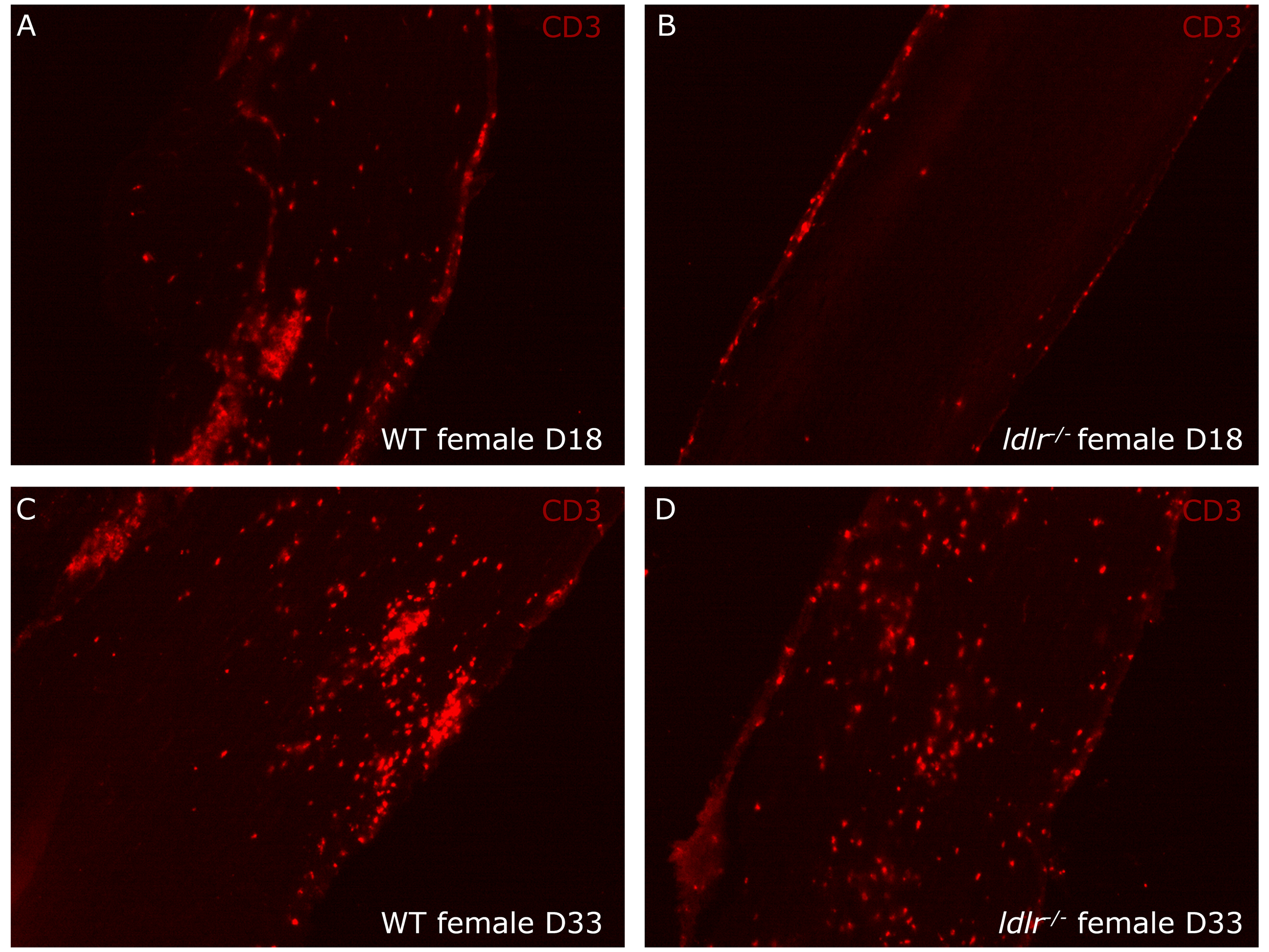

Supplement: Figure S2 — Fluorescent staining of T cells (CD3) in experimental autoimmune encephalomyelitis spinal cord. Staining of T cells in wild-type female (A,C) and ldlr−/− female mice (B,D) on day 18 and 33, respectively. [file Image_2.tif]
